# Upregulated SAE1 Drives Tumorigenesis and Is Associated with Poor Clinical Outcomes in Breast Cancer

**DOI:** 10.1155/2024/2981722

**Published:** 2024-06-30

**Authors:** Hong Liu, Jing Wang, Yunhai Li, Feng Luo, Lei Xing

**Affiliations:** ^1^ Department of Breast and Thyroid Surgery The First Affiliated Hospital of Chongqing Medical University, No. 1 Youyi Rd, Chongqing 400016, China; ^2^ Chongqing Key Laboratory of Molecular Oncology and Epigenetics The First Affiliated Hospital of Chongqing Medical University, No. 1 Youyi Rd, Chongqing 400016, China; ^3^ Department of Head Neck and Breast Surgery The First Affiliated Hospital of USTC Division of Life Sciences and Medicine University of Science and Technology of China, Hefei 230031, Anhui, China; ^4^ Department of Head Neck and Breast Surgery Anhui Provincial Cancer Hospital, Hefei 230031, Anhui, China

## Abstract

**Background:**

The purpose of this study was to analyze SUMO activating enzyme subunit 1 (SAE1) expression in breast cancer (BC). Through bioinformatics analysis and in vitro experiments, the biological function and possibly associated signal pathways of SAE1 in BC were further analyzed.

**Methods:**

Bioinformatics analysis was applied to analyze SAE1 expression in BC and normal breast tissues, its relationship with clinicopathologic characteristics and prognosis in BC patients, and data from the Cancer Genome Atlas database and Gene Expression Omnibus dataset. We performed immunohistochemistry to analyze SAE1 expression in BC tissues and para-cancer tissues in 79 breast cancer patients. BC cell proliferation was detected with the Cell Counting Kit-8 and by the colony formation assay. Cell cycle progression was analyzed by flow cytometry, and the expression of cell cycle-related proteins (E2F1, cyclin D3, and cyclin-dependent kinase 2) was determined by western blots in SAE1 small interfering RNA (siRNA) transfected cells. The GSE1456 dataset was used to analyze possible signal pathways associated with SAE1 by gene set enrichment analysis (GSEA), and the expression of PI3K/AKT/mTOR pathway-related proteins (such as p-PI3K, p-AKT, and mTOR) in SAE1-siRNA cells was detected by western blots.

**Results:**

The bioinformatics and immunohistochemical results showed that SAE1 mRNA and protein expression in BC tissues were significantly higher than those in normal tissues. The SAE1 overexpression was significantly associated with the tumor size, tumor-node-metastasis stage, estrogen receptor, progesterone receptor, human epidermal growth factor receptor 2, and whether or not it was a triple-negative BC. Patients with SAE1 overexpression had a worse overall survival (OS), recurrence-free survival (RFS), and distant metastasis-free survival compared with lower expression patients. Multivariate Cox regression analysis showed that SAE1 may be an independent prognostic factor for OS of BC patients. The proliferation and cell cycle process of BC cells were inhibited by SAE1-siRNA in vitro. The result of GSEA showed that SAE1 was significantly associated with 12 gene sets, including unfolded protein reaction, DNA repair, oxidative phosphorylation, and cell cycle, among others. Additionally, two signal pathways, mTORC1 and PI3K/Akt/mTOR, were significantly correlated with SAE1 overexpression. Western blots confirmed that the expression of PI3K/Akt/mTOR pathway-related proteins (p-PI3K, p-AKT, and mTOR) in BC cells was decreased after knocking down SAE1.

**Conclusion:**

SAE1 was highly expressed in BC. Its overexpression was associated with poor BC prognosis. Additionally, it was an independent prognostic factor for BC patients. We demonstrated that in vitro SAE1 knockdown effectively inhibited BC proliferation and its cell cycle process. Furthermore, the biological function of SAE1 may be associated with the PI3K/Akt/mTOR pathway. SAE1 will be a potential target for BC treatment.

## 1. Introduction

In 2020, with approximately 2.3 million new cases, breast cancer (BC) has globally become the most common malignant tumor. It is also the main cause globally of cancer mortality in women [1]. In China, according to the data of GLOBOCAN 2020, the estimated age-standardized incidence rate of female breast cancer was 39.10/100000 in 2020. Breast cancer is one of the most harmful malignant tumors, among which it currently has the highest morbidity rate in China [2]. As a heterogeneous tumor, breast cancer is frequently divided into the following molecular subtypes: Luminal A, Luminal B, human epidermal growth factor receptor 2 (HER2) overexpression, and triple-negative breast cancer (TNBC), according to the expression of estrogen receptor (ER), progesterone receptor (PR), HER2, and proliferation marker Ki67 [3]. Although the development of precision medicine and individualized treatment for breast cancer patients has greatly improved the prognosis of these patients, many early breast cancers still become advanced breast cancers which cannot be cured [4].

Post-translational protein modification (PTM), including phosphorylation, acetylation, ubiquitination, and sumoylation, is one of the most important regulatory mechanisms of cellular proteins. PTM can alter the activity, intracellular distribution, protein interactions, and lifespan of target proteins [5]. Protein modification by small ubiquitin-like modifier (SUMO), also known as sumoylation, is a reversible post-translational protein modification which occurs in almost all eukaryotes. It is critical to maintain genomic integrity, regulator gene expression, and intracellular signal transduction and also plays an important role in tumor occurrence and development. Sumoylation regulates many biological processes, including DNA damage repair, immune response, tumorigenesis, cell cycle progression, and apoptosis [6, 7]. It requires the involvement of various enzymes, including SUMO-activating enzyme E1 (SUMO E1), SUMO-conjugating enzyme E2, and SUMO-ligating enzyme E3. SUMO E1 is a heterodimer of SUMO activating enzyme subunit 1 (SAE1) and SUMO activating enzyme subunit 2 [6, 8]. SAE1 is highly expressed in various malignant tumors and is closely associated with the tumorigenesis and tumor development. It is overexpressed in hepatocellular carcinoma and is associated with cancer metastasis, disease progression, and poor prognosis [9]. SAE1 is highly expressed in colon cancer cells, and inhibiting SAE1 leads to cell cycle arrest, cell apoptosis, and inhibition of cell proliferation in colon cancer cells [10]. It is upregulated in glioma and promotes glioma cell proliferation and migration by increasing the sumoylation and phosphorylation of protein kinase B (PKB/AKT), leading to glioma development in vitro and in vivo [11]. There is also literature of bioinformatics data and clinical specimen verification that SAE1 is highly expressed in TNBC and is associated with patient prognosis [12]. However, the possible mechanism of SAE1 affecting the biological function of breast cancer cells and the prognosis of patients with breast cancer has, to date, not been reported.

In this study, the Cancer Genome Atlas (TCGA), Gene Expression Omnibus (GEO), and Kaplan-Meier plotter databases were applied to analyze SAE1 expression in breast cancer and its relationship with patient prognosis. We also investigated the relationship between SAE1 and the clinicopathologic characteristics of breast cancer patients through clinical samples. The biological function of SAE1 was also studied in breast cancer cells in vitro. Finally, we can now better understand the role of SAE1 in the tumorigenesis and development of breast cancer and thus its possible value in breast cancer diagnosis, treatment, and prognosis evaluation.

## 2. Materials and Methods

### 2.1. Human BC Tissue Specimens

The BC tissue and adjacent breast tissue specimens used in this study were provided by BC patients who underwent surgical resection and had no history of chemotherapy or immunotherapy prior to surgery at the First Affiliated Hospital of Chongqing Medical University (Chongqing, China). Tissue samples were immediately immersed in liquid nitrogen after resection. All patients signed their informed consent forms, and the study was authorized by the Ethics Committee of the First Affiliated Hospital of Chongqing Medical University (approval ID: 2022-K221). All methods were carried out in accordance with the institutional guidelines and regulations.

### 2.2. TCGA and GEO Dataset Analyses

The TCGA data were sourced from the UCSC database (version 2015-02-24, https://genomecancer.ucsc.edu/). The GEO dataset (GSE42568) was sourced from the GEO database (https://www.ncbi.nlm.nih.gov/geo/). In the TCGA database, 1,095 breast cancer samples and 113 normal tissue samples were used to analyze the difference in SAE1 expression between cancer tissues and normal tissues, and 954 breast cancer patients were used to analyze the relationship between SAE1 and clinicopathologic characteristics of BC patients.

### 2.3. Cell Lines and Culture Conditions

MCF-10A (American Type Culture Collection, ATCC, USA) and MB-468 (ATCC, USA) cells were cultured in MEGM BulletKit (Lonza, Basel, Switzerland) and DMEM medium (Gibco, Carlsbad, CA, USA), respectively. T47D (ATCC, USA), MDA-MB-231 (ATCC, USA), and BT-549 (ATCC, USA) cells were cultured in RPMI 1640 medium (Gibco). The cell media contained 10% fetal bovine serum, 100 U/mL penicillin, and 100 mg/mL streptomycin. Cells were cultured in a humidified incubator (5% CO2, 37°C).

### 2.4. Antibodies

The antibody for immunohistochemistry (IHC) was anti-SAE1 (Abcam, ab185552). The following antibodies were used for western blotting (WB): anti-E2F1 (Abcam), anti-cyclin D3 (Abcam), anti-cyclin-dependent kinase 2 (anti-CDK2) (Abcam), anti-SAE1 (Abcam, ab185552), anti-*β*-actin (Abcam, ab8226), anti-p-PI3K (Abcam, ab182651), anti-AKT (Abcam, ab179463), anti-p-AKT (Abcam, ab192623), and anti-mTOR (Abcam, ab134903).

### 2.5. IHC

All specimens were used to produce 4 *μ*m paraffin sections. For IHC analysis, following deparaffinization and rehydration, antigen retrieval was performed by microwaving the slides for 20 min in sodium citrate-hydrochloric acid buffer solution at 95°C. Endogenous horseradish peroxidase (HRP) activity was blocked with 3% hydrogen peroxide at room temperature. Following three washes with phosphate-buffered saline (PBS), the slides were blocked with normal goat serum and incubated with primary antibodies overnight at 4°C. Subsequently, the slides were incubated with secondary antibodies at 37°C (1 h) followed by HRP-labeled streptavidin solution (10 min), stained with diaminobenzidine, and counterstained with hematoxylin. IHC scores were determined by the staining intensity (negative: 0; weak: 1; moderate: 2; strong: 3) and the percentage of positive cells (<5%: 0; 5%–25%: 1; 26%–50%: 2; 51%–75%: 3; >75%: 4). An overall score was derived by multiplying the intensity and percentage scores.

### 2.6. Western Blot Analysis

Total protein of breast cancer cells was exacted with RIPA lysis buffer and quantified by the BCA assay. The total protein was separated with a 10% SDS-PAGE gel and transferred to PVDF membranes (Bio-Rad, Hercules, CA, USA). The membranes were blocked in 5% non-fat milk for 1 h at room temperature and incubated with primary antibodies overnight at 4°C. The membranes were subsequently incubated with secondary antibodies for 1 h at room temperature. Images of the bands were visualized using an ECL chemiluminescence system (Kodak, Tokyo, Japan).

### 2.7. Gene Set Enrichment Analysis (GSEA)

GSEA was performed with the Broad Institute GSEA software 3.0. The gene expression data of 159 breast cancer cases in the GSE1456 were downloaded from the GEO database. Patients were divided into SAE1-high (*n* = 79) and SAE1-low (*n* = 80) groups based on the median SAE1 expression (median value = 7.856). The gene set “C1. Hallmark” was downloaded from the Molecular Signatures Database (https://software.broadinstitute.org/gsea/msigdb/index.jsp) and was used for the enrichment analysis. A false discovery rate <0.25 and normal *P* value <0.05 were considered to be significantly enriched.

### 2.8. Small Interfering RNA (siRNA) and Transfection

All siRNAs including the negative control siRNA were synthesized by Sangon Biotech (Sangon Biotech (Shanghai) Co., Ltd., Shanghai, China). The sequences of siRNAs targeting SAE1 were as follows: si-SAE1-1, sense: CUCUUAAAGUUCCGUACAGAUTT and antisense: AUCUGUACGGAACUUUAAGAGTT, si-SAE1-2, sense: GAACAGGUAACUCCAGAAGAUTT and antisense: AUCUUCUGGAGUUACCUGUUCTT, si-SAE1-3, sense: GCAUGAGUUUGUAGAGGAGAATT and antisense: UUCUCCUCUACAAACUCAUGCTT, and si-NC, sense: UUCUCCGAACGUGUCACGUTT and antisense: ACGUGACACGUUCGGAGAATT. T47D and BT-549 cells were transfected with siRNA and transfection reagent according to the manufacturer's instructions.

### 2.9. Cell Proliferation and Cell Cycle Assays

The colony formation and Cell Counting Kit-8 (CCK-8) assays were applied to detect the proliferative capabilities of breast cancer cells. For the colony formation assay, transfected cells were inoculated into a 12-well plate at 1,000 cells/well. After 1 week of cell culture, cells were then fixed with 4% paraformaldehyde for 10 min and stained with Crystal Violet. For the CCK-8 assay, 100 *μ*L of cells (1 × 10^4^/mL) from each group was inoculated into 96-well plates. Cell viability was measured at the indicated time points using the CCK-8 (Bosterbio, Wuhan, China) according to the manufacturer's protocols. For the cell cycle assays, cells were collected and fixed in ice-cold ethanol (70%) at 4°C overnight. Cell cycle analysis was subsequently implemented after centrifuging and washing with PBS by PI staining with a flow cytometer (BD Biosciences, Franklin Lakes, NJ, US).

### 2.10. Statistical Analysis

Statistical analyses were conducted using GraphPad Prism 5.0 software (San Diego, CA, USA) and IBM SPSS 22.0 software (Armonk, NY, USA). Student's *t*-tests and chi-square tests were applied to examine the statistical relevance between groups and the correlation between SAE1 expression and clinicopathologic characteristics of BC patients, respectively. Kaplan–Meier analysis was performed to plot survival curves, which were tested by the log-rank test. The prognostic significance of SAE1 was evaluated by univariate and multivariate Cox regression analyses. A *P* value <0.05 was regarded as being statistically significant.

## 3. Results

### 3.1. SAE1 Is Overexpressed in Breast Cancer and Is Associated with Clinicopathologic Characteristics and Poor Prognosis of BC Patients

**Figure 1:**
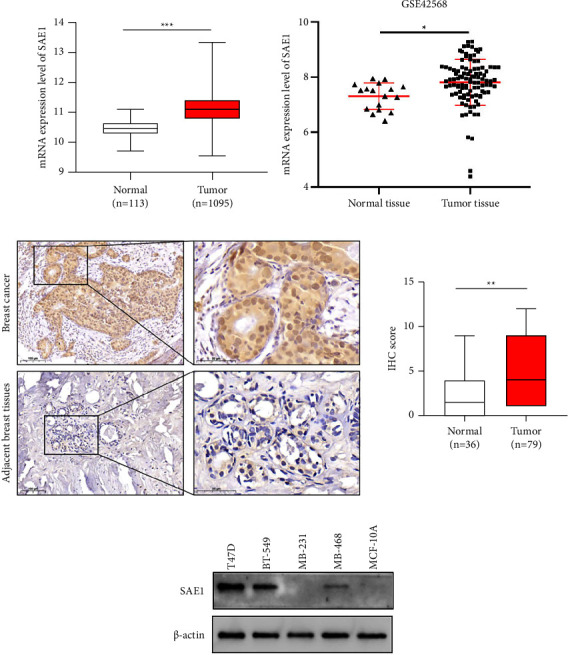
Expression of SAE1 in breast cancer. (a) The mRNA expression levels of SAE1 in 1095 breast cancer tissues and 113 normal tissues from TCGA database. (b) The mRNA expression levels of SAE1 in 104 breast cancer tissues and 17 normal tissues from GSE42568. (c) The protein expression levels of SAE1 in breast cancer tissues (*n* = 79) and adjacent non-tumor tissues (*n* = 36) detected by IHC. (d) The protein expression levels of SAE1 in normal and breast cancer cell lines. The data are presented as mean ± SD. ^*∗*^*p* < 0.05; ^*∗∗*^*p* < 0.01; ^*∗∗∗*^*p* < 0.001.

To verify SAE1 expression in breast cancer tissues and normal breast tissues, we analyzed data of SAE1 mRNA expression in 1095 breast cancer tissues and 113 normal tissues from the TCGA database and in 104 breast cancer tissues and 17 normal tissues from the GEO dataset GSE42568. The results showed that the SAE1 mRNA expression level in breast cancer was significantly higher than that in normal tissue (Figures 1(a) and 1(b)). We also verified the SAE1 protein level in 79 breast cancer tissues and 36 para-cancerous tissues by IHC. The results showed that SAE1 protein expression in breast cancer tissues was significantly higher than that in para-cancerous tissues (Figure 1(c)). Subsequently, WB was performed to detect SAE1 expression in normal breast epithelial cell (MCF-10A) and some breast cancer cell lines. The results showed that SAE1 was significantly overexpressed in T47D and BT-549 cells (Figure 1(d)).

**Figure 2:**
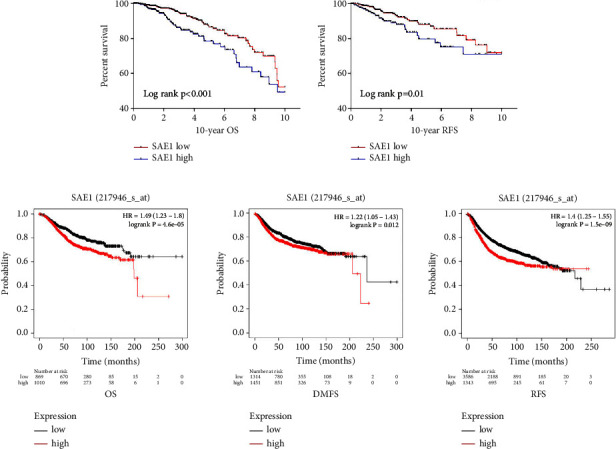
Survival analysis of the relationships between SAE1 expression and clinical outcomes in Kaplan-Meier plotter database and TCGA database. (a) Survival curves showing the association of SAE1 with OS and RFS in TCGA cohort. (b) Kaplan–Meier analysis of the relationships between SAE1 expression and clinical outcomes in Kaplan-Meier plotter database.

Subsequently, we analyzed the correlation between the SAE1 expression level and clinicopathologic characteristics (cutoff value 11.2) in 954 breast cancer patients (data from the TCGA database) and found that SAE1 overexpression was significantly associated with the tumor size (*P* < 0.001), tumor-node-metastasis (TNM) staging (*P* = 0.002), ER (*P* < 0.001), PR (*P* < 0.001), HER2 (*P* < 0.001), and whether or not it was TNBC (*P* < 0.001) (Table 1). By plotting survival curves, it was found that among these patients, those with SAE1 overexpression had a shorter overall survival (OS) and recurrence-free survival (RFS) (Figure 2(a)). In terms of subgroup analysis of these patients according to ER, PR, HER2, and whether or not it was TNBC, we found that in patients who were ER negative, PR negative, HER2 negative, or TNBC, those with SAE1 overexpression had a shorter OS. However, there was no significant difference in the OS among patients who were ER positive, PR positive, HER2 positive, or non-TNBC patients (Supplementary Figure S1). Similarly, in the Kaplan-Meier plotter database, patients with SAE1 overexpression had shorter OS, RFS, and distant metastasis-free survival (DMFS) compared to the patients with low SAE1 expression (Figure 2(b)). Multivariate Cox regression analysis showed that SAE1 may be an independent prognostic factor for the OS of breast cancer (Table 2).

### 3.2. Knocking Down SAE1 Inhibits Breast Cancer Cell Proliferation and Cell Cycle Progression

**Figure 3:**
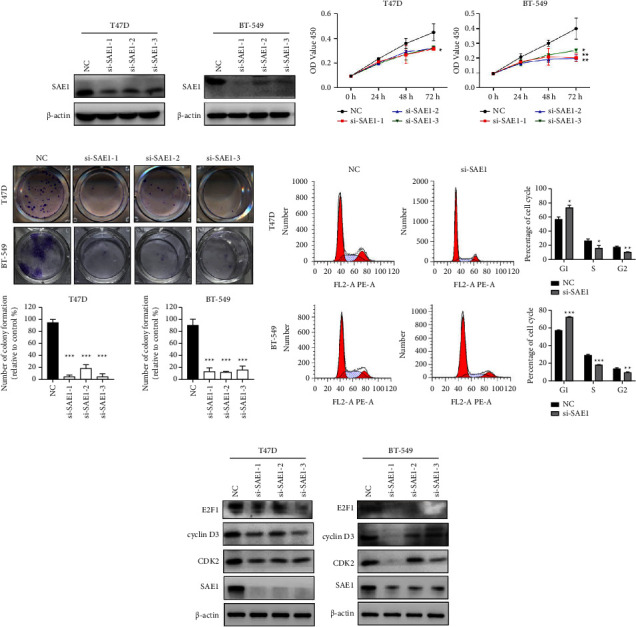
SAE1-siRNA inhibits proliferation and cell cycle progression in breast cancer cells. (a) Relative expression of SAE1 was determined in T47D and BT-549 cells transfected with siRNA by WB. (b) The cell viability was measured in T47D and BT-549 cells transfected with siRNA by CCK-8 assay. (c) Cell survival was evaluated in T47D and BT-549 cells transfected with siRNA by colony formation assay. Transfected cells were inoculated into a 12-well plate at 1,000 cells/well. (d) The cell cycle of T47D and BT-549 cells transfected with siRNA detected by flow cytometry. (e) The cell cycle-related proteins were detected in T47D and BT-549 cells transfected with siRNA by WB. Data are presented as mean ± SD. ^*∗*^*p* < 0.05; ^*∗∗*^*p* < 0.01; ^*∗∗∗*^*p* < 0.001.

To investigate the biological function of SAE1 in breast cancer cells (T47D, BT-549), we knocked down the SAE1 expression level in cells by transfecting with siRNA (Figure 3(a)). The CCK-8 assay showed that SAE1 knockdown significantly inhibited T47D and BT-549 cell growth (Figure 3(b)). The colony formation assay further demonstrated that downregulating SAE1 significantly inhibited T47D and BT-549 cell proliferation (Figure 3(c)). The cell cycle assays showed that compared with the negative control (NC) group, the proportion of G1 phase cells increased, whereas that of the S and G2 phases decreased in the SAE1-siRNA-treated T47D and BT-549 cells (Figure 3(d)). Subsequently, several cell cycle-related proteins were examined by WB. This showed that E2F1, cyclin D3, and CDK2 expression were also decreased in SAE1-siRNA-treated T47D and BT-549 cells compared with the NC group (Figure 3(e)). These results indicated that SAE1 knockdown inhibited breast cancer cell growth in vitro.

### 3.3. The Biological Functions and Related Signal Pathways of SAE1 in BC

**Figure 4:**
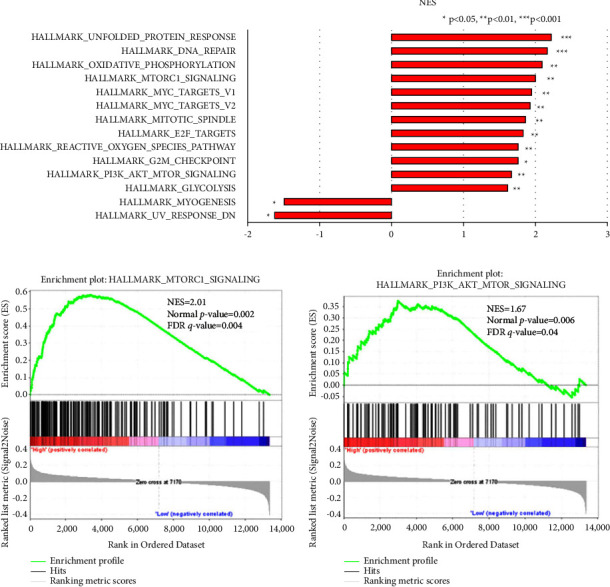
Gene set enrichment analysis related with SAE1. Gene enrichment plots showed that a series of gene sets including (a) 12 gene sets were related to SAE1 overexpression. (b) HALLMARK_MTORC1_SIGNALING. (c) HALLMARK_PI3K_AKT_MTOR_SIGNALING.

**Figure 5:**
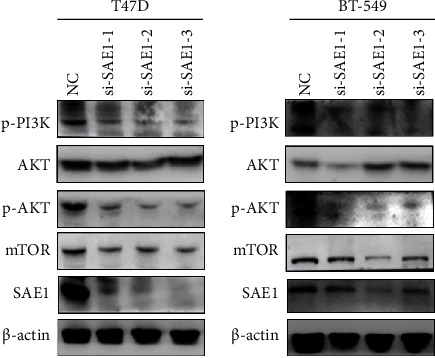
The PI3K/Akt/mTOR signal pathway-related proteins were detected in T47D and BT-549 cells transfected with siRNA by WB.

To further analyze the potential biological functions and related signal pathways of SAE1 in breast cancer, GSEA was performed with the GEO dataset GSE1456. In total, 159 cases of breast cancer patients were divided into high and low expression groups based on SAE1 expression levels (Supplementary Figure S2). The results showed a significant association between SAE1 and 14 gene sets. Among these, 12 gene sets were related to SAE1 overexpression, including unfolded protein reaction, DNA repair, oxidative phosphorylation, and cell cycle, among others (Figure 4(a)). At the same time, there were two signaling pathways, mTORC1 and PI3K/Akt/mTOR, that were significantly associated with SAE1 overexpression (Figures 4(b) and 4(c)). Consequently, WB was performed to detect the expression of PI3K/Akt/mTOR signaling pathway-related proteins. The results showed that p-PI3K, p-AKT, and mTOR expressions were decreased in SAE1-siRNA treated T47D and BT-549 cells compared with the NC group (Figure 5). These results suggested that the effect of SAE1 on breast cancer cells may be associated with the PI3K/Akt/mTOR pathway.

## 4. Discussion

Sumoylation is involved in the tumorigenesis and development of various cancers, and sumoylation of different proteins may promote or inhibit tumor progression. The 5-methylcytosine RNA methyltransferase NSUN2, which is upregulated and involved in cell proliferation and metastasis in various cancers, can enhance the carcinogenic ability by being stabilized by sumoylation [13]. In lung cancer, HIF-1 *α* sumoylation can promote invasion and metastasis [14]. Sumoylation of mesencephalic astrocyte-derived neurotrophic factor inhibits the NF-*κ*B/Snail signaling pathway and epithelial mesenchymal transition, thereby inhibiting the invasion and metastasis of liver cancer [15].

The process of sumoylation requires the participation of multiple enzymes. SAE1, as a subunit of SUMO E1, plays an important role in sumoylation. It is overexpressed in various tumors, promoting tumor progression and significantly correlating with the prognosis of cancer patients [9, 11, 16]. Furthermore, inhibiting SAE1 expression can effectively inhibit tumorigenesis and tumorous development [10, 16, 17]. In the present study, both bioinformatics analysis of the TCGA and GEO databases and immunohistochemical assay of clinical tissue samples showed that SAE1 was highly expressed in breast cancer. Moreover, SAE1 overexpression significantly correlated with poor OS, RFS, and DMFS of breast cancer patients. Multivariate Cox regression analysis showed that SAE1 may be an independent prognostic factor for the OS of breast cancer patients. Additionally, SAE1 expression was significantly associated with the tumor size, TNM stage, ER, PR, and HER2 expression, and whether or not it was TNBC. The gene enrichment analysis in this study showed that SAE1 overexpression was associated with 12 gene sets, including unfolded protein reaction, DNA repair, oxidative phosphorylation, and cell cycle, among others. The SAE1 protein expression varies among different breast cancer cell lines and normal mammary cell lines. The SAE1 expression is absent in MB-231. We speculate that SAE1 may be mutated in MB-231, but we did not find evidence to support this by searching the literature. We used siRNA to knock down SAE1 expression in T47D and BT-549 cells, and the results showed that this knockdown significantly inhibited breast cancer cell proliferation and the cell cycle process.

In lung adenocarcinoma and glioma, studies have shown that SAE1 expression upregulation promoted AKT sumoylation, further promoting AKT phosphorylation, thereby activating the AKT signaling pathway [11, 17]. In hepatocellular carcinoma, SAE1 expression is also upregulated, which promotes cancer cell proliferation, invasion, and metastasis by enhancing mTOR sumoylation [16]. Our GSEA result showed that two signaling pathways, mTORC1 and PI3K/Akt/mTOR, were significantly correlated with SAE1 overexpression. PI3K/Akt/mTOR is a signaling pathway that plays a crucial role in tumorigenesis, cancer development, and cancer treatment [18, 19]. Our WB experiment confirmed that knocking down SAE1 resulted in decreased p-PI3K, p-AKT, and mTOR expressions which were associated with the PI3K/Akt/mTOR signaling pathway. These results suggested that the effect of SAE1 on breast cancer may be through the PI3K/Akt/mTOR signaling pathway. In breast cancer, enhancement of the PI3K/Akt/mTOR signaling pathway promotes cell proliferation and inhibits cell apoptosis. In addition, targeted drugs of this signaling pathway are gradually increasing in the basic research and the clinical treatment of breast cancer [20, 21]. For example, alpelisib, as a drug targeting PI3K*α*, combined with fulvestrant are used to treat advanced or metastatic postmenopausal female or male breast cancer (whose tumor is HR positive, HER2 negative, and with the PIK3CA mutation) [22]. Everolimus, as a drug targeting mTOR, combined with exemestane are used to treat HR-positive and HER2-negative advanced or metastatic breast cancer (who have previously received letrozole and anastrozole) [23]. Similarly, blocking tumor growth-promoting sumoylation through inhibitors also has certain prospects in tumor therapy [24]. Our study showed that the proliferation and cell cycle progression of breast cancer cells were significantly inhibited after SAE1 inhibition in vitro.

## 5. Conclusions

SAE1 was highly expressed in breast cancer, and its overexpression was associated with poor prognosis of breast cancer patients. Knocking down SAE1 effectively inhibited breast cancer cell proliferation and its cell cycle process. The biological function of SAE1 may be achieved through the PI3K/Akt/mTOR signaling pathway, and SAE1 is a potential target for breast cancer treatment.

## Figures and Tables

**Table 1:** Correlation of SAE1 expression level with the clinicopathological features in 954 breast cancer patients of TCGA cohort.

Characteristics	Number of cases	SAE1		
		Low (*n*)	High (*n*)	*P* value
*Age*				
<55	398	236	162	0.64
≥55	556	339	217	

*Anatomic subdivision*				
Left	496	285	211	0.07
Right	458	290	168	

*Tumor size*				
T1	249	178	71	<0.001^*∗*^
T2	554	316	238	
T3	121	70	51	
T4	30	11	19	

*Lymph node metastasis*				
N0	457	285	172	0.22
N1	328	191	137	
N2	102	65	37	
N3	67	34	33	

*TNM stage*				
I	169	118	51	0.002^*∗*^
II	550	324	226	
III	222	130	92	
IV	13	3	10	

*ER*				
Positive	732	486	246	<0.001^*∗*^
Negative	222	89	133	

*PR*				
Positive	639	432	207	<0.001^*∗*^
Negative	315	143	172	

*HER2*				
Positive	184	87	97	<0.001^*∗*^
Negative	770	488	282	

*Triple negative breast cancer*				
Yes	166	62	104	<0.001^*∗*^
No	788	513	275	

^*∗*^ *p* < 0.05 was considered statistically significant.

**Table 2:** Univariate and multivariate Cox regression analysis of SAE1.

Variants	Univariate analysis			Multivariate analysis		
	HR	95% CI	*p* value	HR	95% CI	*p* value
Age (<55 vs. ≥55)	1.91	1.25–2.92	0.003^*∗*^	2.08	1.35–3.21	0.001^*∗*^
Tumor size						
T1	Reference			Reference		
T2	1.51	0.90–2.56	0.12	1.31	0.76–2.26	0.34
T3	1.37	0.69–2.70	0.37	1.01	0.49–2.09	0.97
T4	3.79	1.74–8.27	0.01^*∗*^	2.16	0.92–5.07	0.08
Lymph node						
N0	Reference			Reference		
N1	1.46	0.91–2.34	0.11	1.33	0.81–2.20	0.26
N2	2.92	1.64–5.20	<0.001^*∗*^	3.34	1.85–6.24	<0.001^*∗*^
N3	3.17	1.51–6.66	0.002^*∗*^	2.69	1.22–5.91	0.01^*∗*^
TNM stage						
I	Reference			NA		
II	1.47	0.76–2.85	0.25	NA		
III	2.95	1.50–5.84	0.02^*∗*^	NA		
IV	8.88	3.66–21.52	<0.001^*∗*^	NA		
ER (negative vs. positive)	1.45	0.94–2.24	0.10	1.03	0.54–1.98	0.93
PR (negative vs. positive)	1.52	1.01–2.28	0.04^*∗*^	1.54	0.84–2.83	0.16
HER2 (negative vs. positive)	0.80	0.48–1.34	0.39	1.07	0.62–1.82	0.82
SAE1 (high vs. low)	1.77	1.19–2.64	0.005^*∗*^	1.46	1.01–2.34	0.04^*∗*^

HR: hazard ratio; CI: confidence interval. Multivariable Cox proportional hazards regression models were adjusted for age, tumor size, lymph node status, ER, PR, and HER2. ^*∗*^*p* < 0.05 was considered statistically significant.

## Data Availability

The original data used in this study are available from the corresponding authors upon reasonable request.

## Supplementary Materials

Supplementary Materials (1) Supplementary Figure S1: survival curves showing the association of SAE1 with OS in the different subgroups of TCGA cohort. (2) Original images for western blot: original, uncropped, and unadjusted images for western blot. (3) Supplementary Figure S2: the mRNA expression levels of SAE1 in GSE1456.
