# Supplementary material for: Upregulated SAE1 Drives Tumorigenesis and Is Associated with Poor Clinical Outcomes in Breast Cancer
Source: Breast J. 2024 Jun 30;2024:2981722. doi: 10.1155/2024/2981722 (PMC11227943; doi:10.1155/2024/2981722)
Supplement: Supplementary Materials — (1) Supplementary Figure S1: survival curves showing the association of SAE1 with OS in the different subgroups of TCGA cohort. (2) Original images for western blot: original, uncropped, and unadjusted images for western blot. (3) Supplementary Figure S2: the mRNA expression levels of SAE1 in GSE1456. [file 2981722.f1.zip › original images for western blot.pdf]

Fig.1D

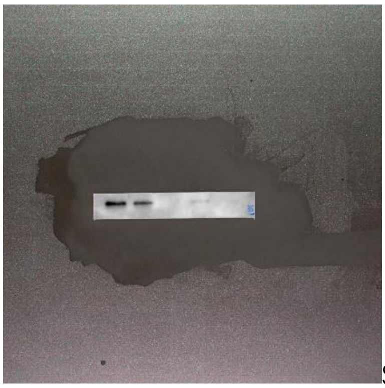

SAE1

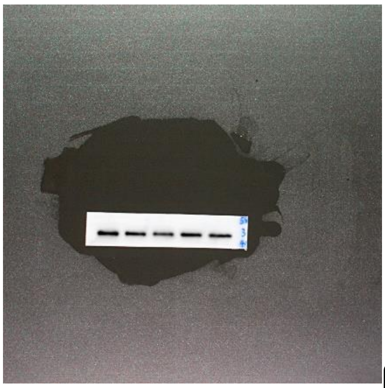

β-actin

fig.3A

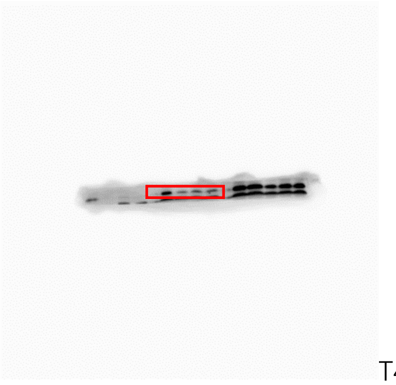

T47D SAE1

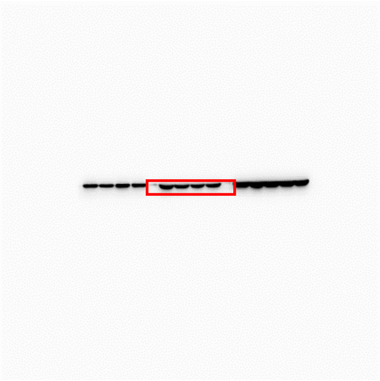

T47D β-actin

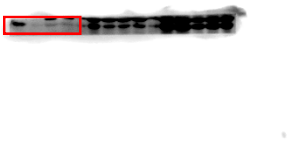

BT-549 SAE1

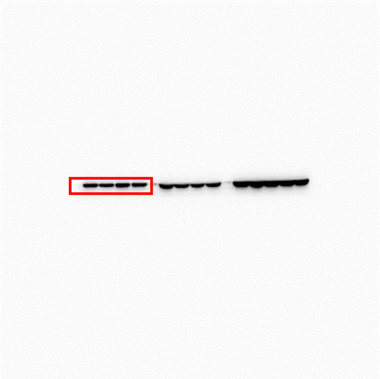

BT-549 β-actin

fig.3E

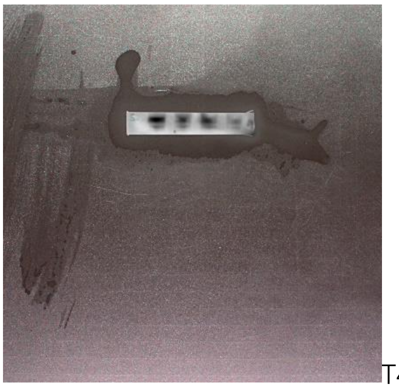

T47D E2F1

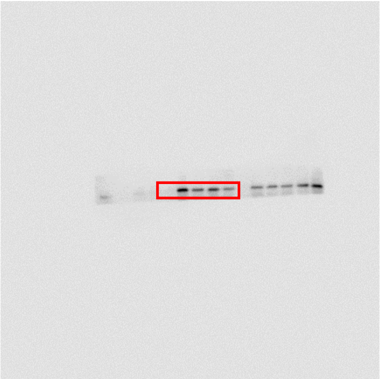

T47D cyclin D3

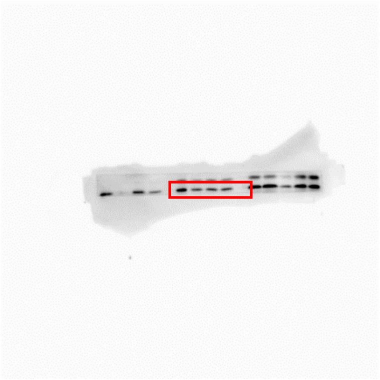

T47D CDK2

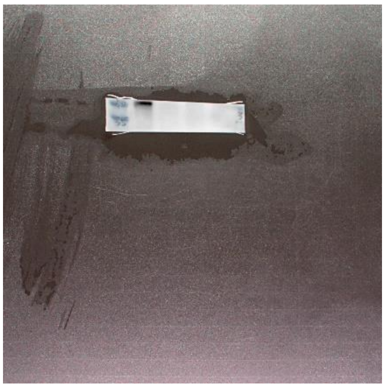

T47D SAE1

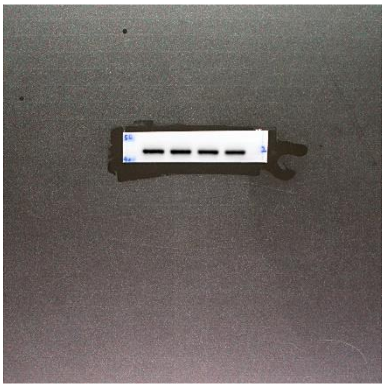

T47D β-actin

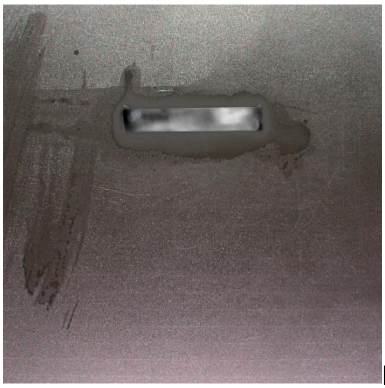

BT549 E2F1

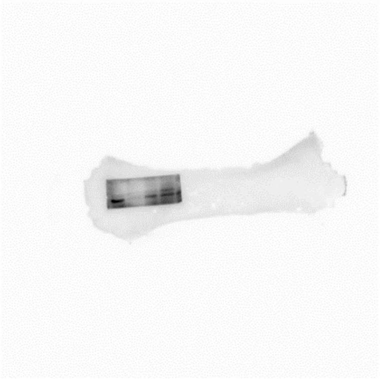

BT549 cyclin D3

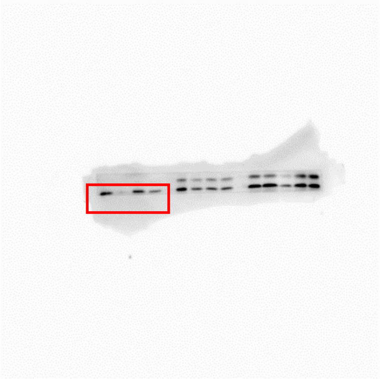

BT549 CDK2

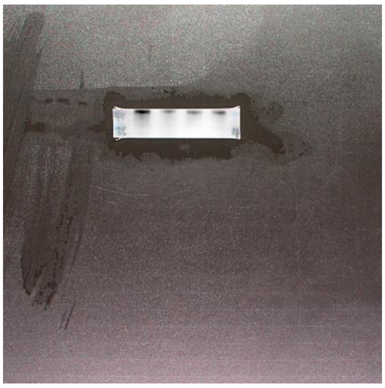

BT549 SAE1

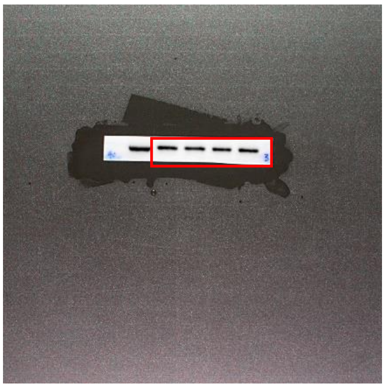

BT549 β-actin

Fig.5

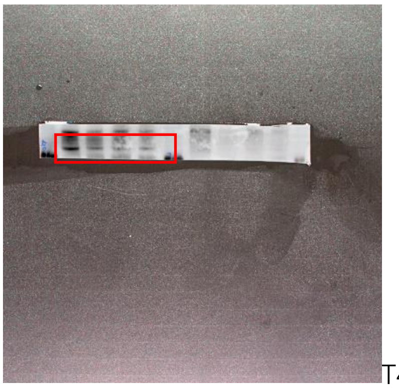

T47D p-PI3K

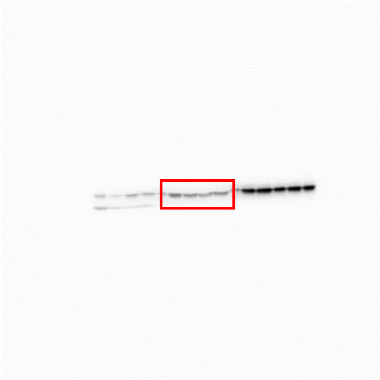

T47D AKT

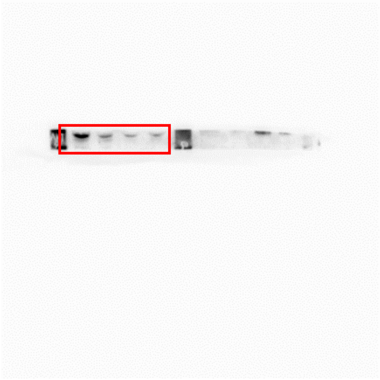

T47D pAKT

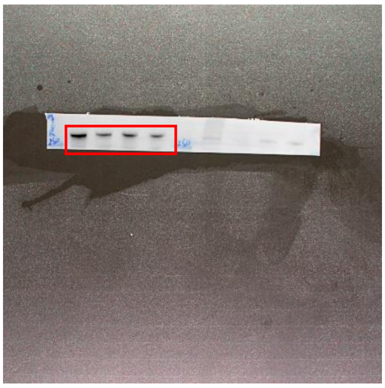

T47D mTOR

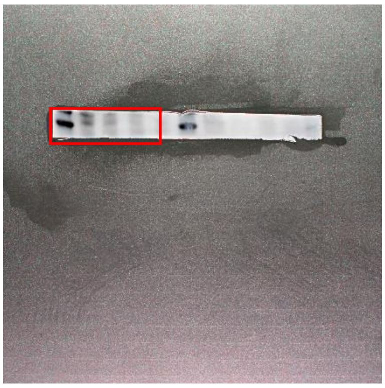

T47D SAE1

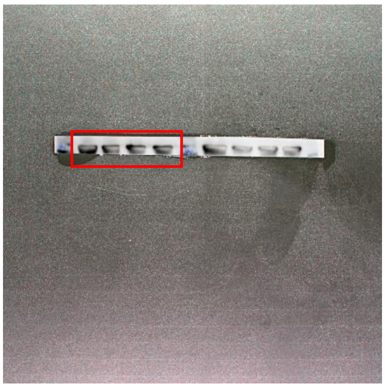

T47D β-actin

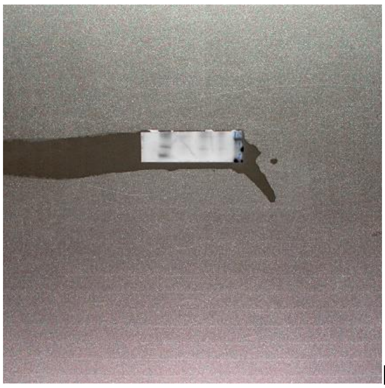

BT-549 p-PI3K

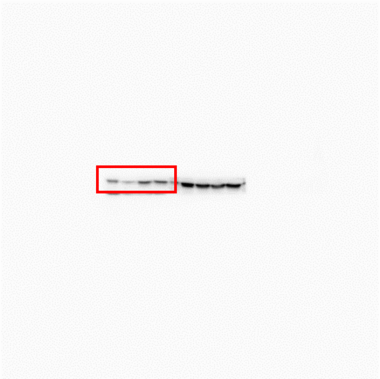

BT-549 AKT

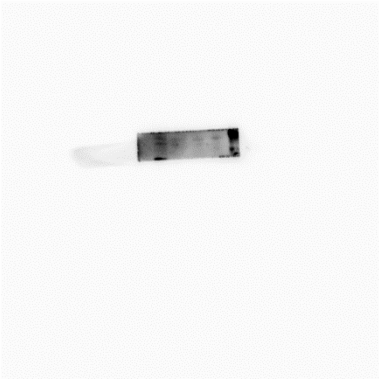

BT-549 p-AKT

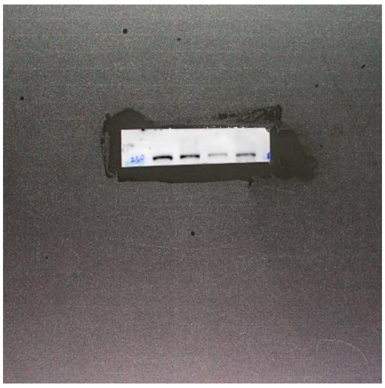

BT-549 mTOR

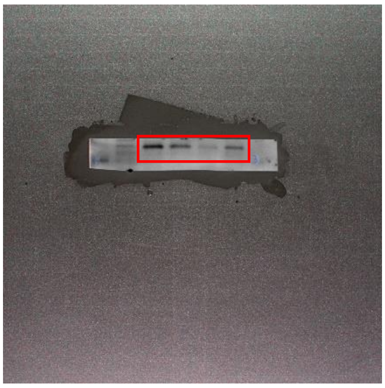

BT-549 SAE1

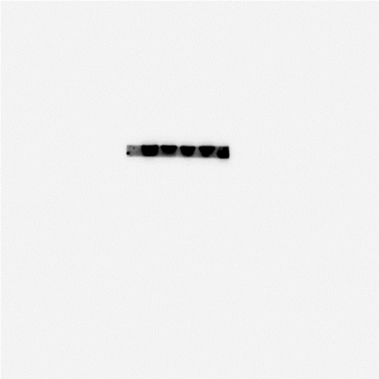

BT-549 β-actin
