# Supplementary figures and images for: Upregulated SAE1 Drives Tumorigenesis and Is Associated with Poor Clinical Outcomes in Breast Cancer
Source: Breast J. 2024 Jun 30;2024:2981722. doi: 10.1155/2024/2981722 (PMC11227943; doi:10.1155/2024/2981722)

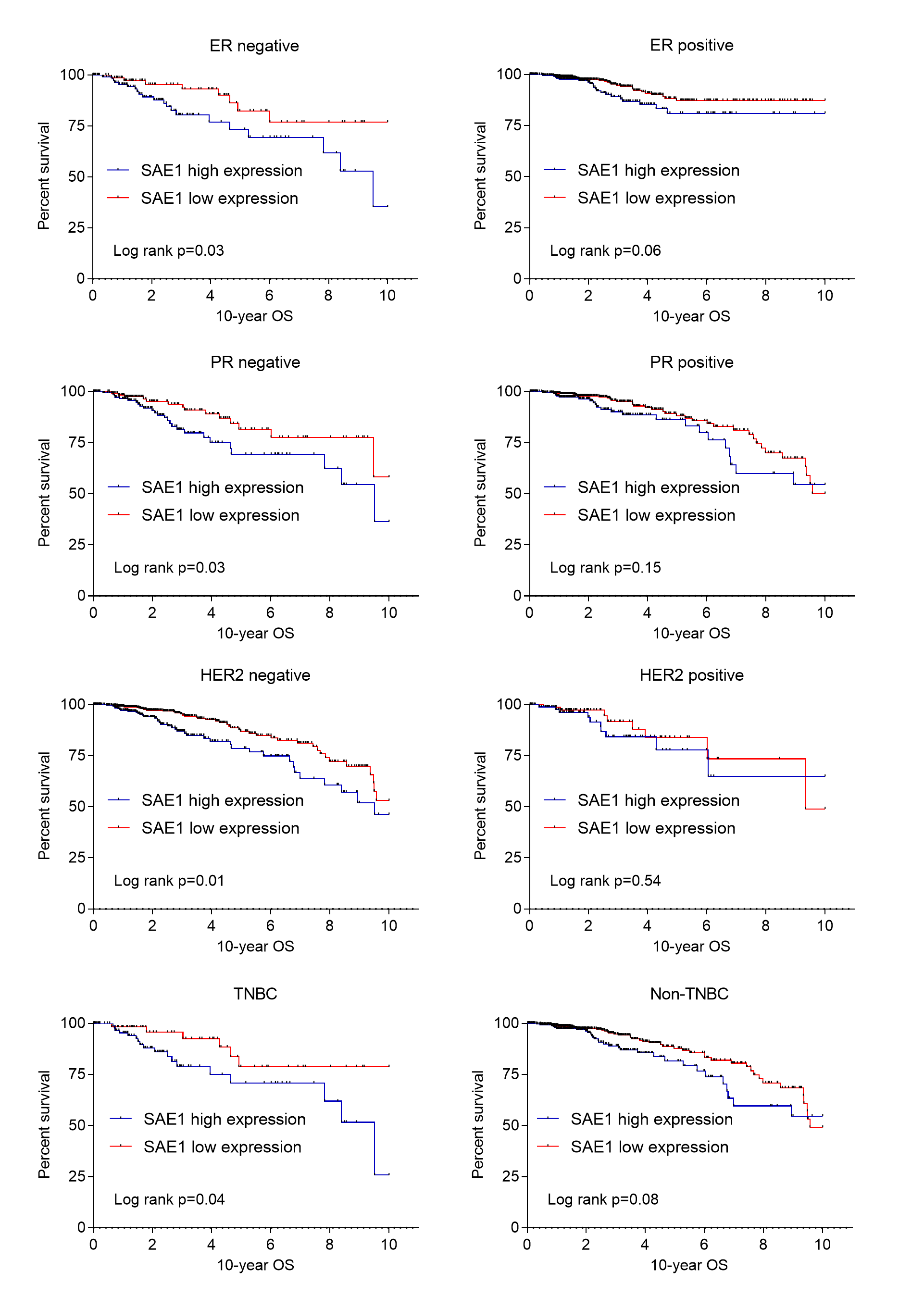

Supplement: Supplementary Materials — (1) Supplementary Figure S1: survival curves showing the association of SAE1 with OS in the different subgroups of TCGA cohort. (2) Original images for western blot: original, uncropped, and unadjusted images for western blot. (3) Supplementary Figure S2: the mRNA expression levels of SAE1 in GSE1456. [file 2981722.f1.zip › Supplementary Figure S1.png]

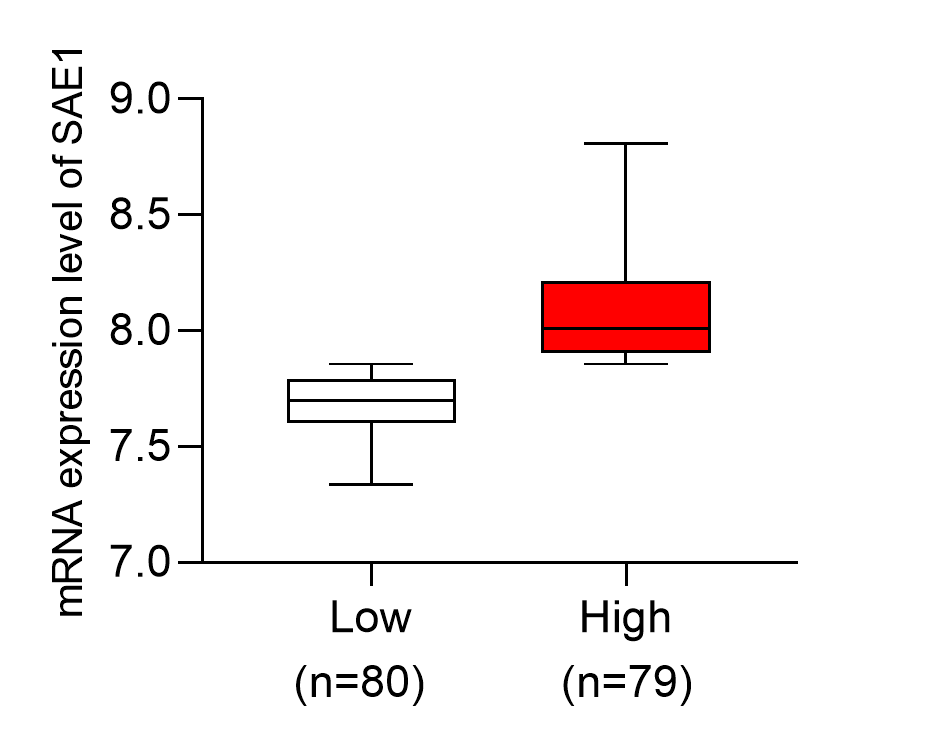

Supplement: Supplementary Materials — (1) Supplementary Figure S1: survival curves showing the association of SAE1 with OS in the different subgroups of TCGA cohort. (2) Original images for western blot: original, uncropped, and unadjusted images for western blot. (3) Supplementary Figure S2: the mRNA expression levels of SAE1 in GSE1456. [file 2981722.f1.zip › Supplementary Figure S2.png]
